# Circulating Tumor DNA Clearance Is Associated with Response and Survival in Metastatic Urothelial Carcinoma Treated with First-Line Enfortumab Vedotin Plus Pembrolizumab

**DOI:** 10.3390/cancers18213532

**Published:** 2026-11-03

**Authors:** Adam Khorasanchi, Sayan Mullick Chowdhury, Eric Fei Hong, Mingjia Li, Changchuan Jiang, Lin Mei, Michael A. Liu, Katharine A. Collier, Ce Shang, Lei Xu, Ashish Manne, Peng Wang, Sofia Jaroniec, Michael Gong, Eric A. Singer, Feng Hong, Harrison Kim, Khalid Niazi, Robert Wesolowski, Mark Rubinstein, Yuanquan Yang, Lingbin Meng

**Affiliations:** 1 Division of Medical Oncology, Department of Internal Medicine, The Ohio State University Comprehensive Cancer Center, Columbus, OH 43210, USA; adam.khorasanchi@osumc.edu (A.K.); sayan.mullickchowdhury@osumc.edu (S.M.C.); mingjia.li@osumc.edu (M.L.); katharine.collier@osumc.edu (K.A.C.); ce.shang@osumc.edu (C.S.); ashish.manne@osumc.edu (A.M.); peng.wang@osumc.edu (P.W.); feng.hong@osumc.edu (F.H.); robert.wesolowski@osumc.edu (R.W.); mark.rubinstein@osumc.edu (M.R.); 2 Olentangy Liberty High School, Powell, OH 43065, USA; erichong0410@gmail.com; 3 Division of Hematology and Oncology, Department of Internal Medicine, University of Texas Southwestern Medical Center, Dallas, TX 75390, USA; changchuan.jiang@utsouthwestern.edu; 4 Abramson Cancer Center, Hospital of the University of Pennsylvania, Philadelphia, PA 19104, USA; lin.mei@pennmedicine.upenn.edu; 5 Division of Hematology and Oncology, Robert H. Lurie Comprehensive Cancer Center, Northwestern University Feinberg School of Medicine, Chicago, IL 60611, USA; michael.liu1@northwestern.edu; 6 Center for Tobacco Research, The Ohio State University Comprehensive Cancer Center, Columbus, OH 43210, USA; lei.xu@osumc.edu; 7 Kenyon College, Gambier, OH 43022, USA; jaroniec1@kenyon.edu; 8 Division of Urologic Oncology, Department of Urology, The Ohio State University Comprehensive Cancer Center, Columbus, OH 43210, USA; michael.gong@osumc.edu (M.G.); eric.singer@osumc.edu (E.A.S.); 9 Department of Radiology, The Ohio State University Comprehensive Cancer Center, Columbus, OH 43210, USA; harrison.kim@osumc.edu; 10 Department of Pathology, The Ohio State University Comprehensive Cancer Center, Columbus, OH 43210, USA; khalid.niazi@osumc.edu

**Keywords:** circulating tumor DNA, metastatic urothelial carcinoma, enfortumab vedotin, pembrolizumab, prognostic biomarker, progression-free survival, overall survival, liquid biopsy

## Abstract

Background/Objectives: Enfortumab vedotin plus pembrolizumab (EV+pembro) is the standard first-line treatment for metastatic urothelial carcinoma (mUC), yet no validated on-treatment biomarker identifies patients deriving a durable benefit. We evaluated whether circulating tumor DNA (ctDNA) clearance is associated with response and survival with this regimen alone. Methods: This single-center retrospective study included 25 patients with mUC receiving first-line EV+pembro. A tumor-informed assay (Signatera) measured ctDNA longitudinally. Responses were assessed by RECIST 1.1. Progression-free survival (PFS) and overall survival (OS) hazard ratios (HRs) were estimated by Cox regression and, to address immortal time bias, with clearance as a time-dependent covariate. Results: Fifteen of 25 patients (60%) achieved ctDNA clearance (ever undetectable). The objective response rate was 76% overall and 100% (15/15) with clearance versus 40% (4/10) without (*p* = 0.0012). Clearance was associated with longer PFS (HR 0.16, 95% CI 0.05–0.54; *p* = 0.003) and OS (HR 0.08, 95% CI 0.01–0.68; *p* = 0.021), with similar Firth penalized HRs (0.17 and 0.11). In the time-dependent analysis, the associations were attenuated (PFS HR 0.35, 95% CI 0.09–1.15, *p* = 0.085; OS HR 0.14, 95% CI 0.01–0.84, *p* = 0.029; profile likelihood CIs). A post-nadir ctDNA rise preceded progression or death by a median of 95 days in four patients (death in two). Conclusions: In this first-line EV+pembro cohort, all patients who cleared ctDNA responded, and clearance was associated with longer survival, although this association was attenuated in the time-dependent analysis. These hypothesis-generating findings support the prospective evaluation of ctDNA-guided monitoring.

## Simple Summary

Cancer of the bladder and urinary tract that has spread is now usually treated with a combination of enfortumab vedotin and pembrolizumab. Many patients benefit, but not all do, and no reliable blood test currently shows early in treatment who is responding. Circulating tumor DNA (ctDNA) is genetic material shed by a tumor into the bloodstream and can be measured with a simple blood draw. We studied 25 patients treated at our center to ask whether changes in ctDNA matched how the cancer responded on scans and how long patients lived. Every patient whose blood test became completely clear responded on imaging, and these patients lived longer before their cancer grew. In some patients, a rise in ctDNA appeared before growth was visible on scans. These findings suggest that blood-based monitoring of ctDNA deserves further study, but they must be confirmed in larger studies before guiding treatment decisions.

## 1. Introduction

Urothelial carcinoma is the most common malignancy of the urinary tract, and patients with locally advanced or metastatic disease have historically faced a poor prognosis [1,2]. For decades, platinum-based chemotherapy was the standard first-line treatment, associated with a median overall survival period of approximately 14–15 months [3,4,5]. Immune checkpoint inhibitors targeting the programmed cell death protein 1 (PD-1)/programmed death ligand 1 (PD-L1) axis represented a major advance, particularly avelumab as switch maintenance therapy after platinum-based chemotherapy [6,7] and pembrolizumab in the platinum-refractory second-line setting [8,9]. More recently, the antibody–drug conjugate enfortumab vedotin (EV), which targets Nectin-4 and delivers the microtubule-disrupting agent monomethyl auristatin E, demonstrated substantial activity as a monotherapy in previously treated disease [10,11]. In combination with pembrolizumab, it produced high response rates in cisplatin-ineligible patients receiving first-line treatment [12]. The phase 3 EV-302/KEYNOTE-A39 trial established enfortumab vedotin plus pembrolizumab (EV+pembro) as the standard first-line treatment for locally advanced or metastatic urothelial carcinoma (mUC), improving progression-free survival (PFS; 12.5 vs. 6.3 months; hazard ratio [HR] 0.45) and overall survival (OS; 31.5 vs. 16.1 months; HR 0.47) relative to platinum-based chemotherapy [13].

Despite this efficacy, responses to EV+pembro are heterogeneous [13,14]. A subset of patients experiences primary or acquired resistance, and no validated on-treatment biomarker reliably identifies patients deriving a durable benefit versus those who may need early treatment modification [14,15]. Radiographic response assessment using the Response Evaluation Criteria in Solid Tumors (RECIST) version 1.1 [16] provides a standardized but intermittent read-out, and imaging can be limited by the delayed detection of progression and by difficulty in distinguishing pseudoprogression from true progression during immunotherapy [17]. Molecular measures providing an earlier, dynamic read-out of disease activity during EV+pembro therapy are therefore needed.

Circulating tumor DNA (ctDNA) is a non-invasive biomarker used across malignancies, enabling the real-time monitoring of the tumor burden and treatment response through a blood draw [18,19,20]. Because ctDNA has a short circulating half-life, on the order of minutes to a few hours, plasma levels are thought to reflect the tumor burden at the time of collection, although shedding also depends on the tumor site and on treatment-induced cell death [18]. In urothelial carcinoma, ctDNA is best validated in the perioperative setting. The phase 3 IMvigor011 trial showed that ctDNA-guided adjuvant atezolizumab improved disease-free survival (DFS; HR 0.64, 95% confidence interval [CI] 0.47–0.87) and OS (HR 0.59, 95% CI 0.39–0.90) in patients who became ctDNA-positive after cystectomy, while patients with persistently undetectable ctDNA had a 2-year DFS rate of 88% without adjuvant therapy [21]. On this basis, the FDA approved ctDNA-guided adjuvant atezolizumab for muscle-invasive bladder cancer with detectable ctDNA after cystectomy in May 2026, with Signatera as the companion diagnostic [22], making ctDNA-guided treatment a standard of care in the perioperative setting. In CheckMate 274, adjuvant nivolumab preferentially benefited patients with detectable ctDNA (DFS HR 0.35, 95% CI 0.18–0.66) compared with those with undetectable ctDNA (HR 0.99, 95% CI 0.51–1.93) [23], and, in the neoadjuvant ABACUS trial, the serial ctDNA status was prognostic at every timepoint, with no relapses among patients who were ctDNA-negative at baseline and after neoadjuvant therapy [24]. Evidence in the metastatic setting is more recent. Reyes and colleagues analyzed 545 plasma samples from 110 patients receiving EV with or without pembrolizumab and found that on-treatment ctDNA positivity was associated with inferior PFS and OS at every interval examined [25]. Karol and colleagues showed that the quantitative baseline ctDNA level carries prognostic information beyond simple detectability, across treatment lines and therapy classes [26]. Aslanova and colleagues reported that a positive baseline result that never converts to negative is associated with poor outcomes in mUC [27]. Müller and colleagues, in a prospective multicenter study of 208 patients, established that ctDNA genotyping identifies actionable *FGFR* alterations that archival tissue testing misses [28]. These studies establish that ctDNA carries prognostic information in metastatic urothelial carcinoma.

Four questions nevertheless remain unanswered. First, none of these cohort studies specifically evaluated the regimen that is now the first-line standard of care for metastatic disease: they included both EV monotherapy and the combination [25,29], spanned multiple treatment lines and therapy classes [26,27], or were designed to compare genotyping platforms rather than to track treatment response [28]. Because ctDNA kinetics reflect the depth and speed of tumor cell killing, the kinetics observed in mixed cohorts cannot confidently be extrapolated to first-line EV+pembro. Second, although on-treatment ctDNA positivity is clearly prognostic, whether achieving undetectable ctDNA identifies a group with longer overall survival under this regimen alone has not been examined: the association was reported in a cohort that combined EV monotherapy and the combination [25], and, in another real-world cohort, overall survival was not evaluable among ctDNA-negative patients [27]. Third, the interval by which a molecular rise precedes radiographic progression rests on a single published estimate of approximately 124 days [29], and this has not been independently replicated. Fourth, the genomic determinants of molecular clearance are largely unexplored: the one real-world series that examined this question found no association between sequencing findings and outcomes [27], and ctDNA genotyping studies have concentrated on identifying targetable alterations rather than on identifying which tumors fail to clear [28]. Consequently, a clinician treating a patient with first-line EV+pembro today cannot say which proportion of patients should be expected to clear ctDNA, whether clearance is associated with longer survival on this regimen, how much warning a rising ctDNA level provides, or which tumors are unlikely to clear.

We therefore studied longitudinal tumor-informed ctDNA in a single-center cohort restricted to patients receiving first-line EV+pembro, with four objectives that mapped onto these gaps: to characterize ctDNA kinetics under a single contemporary regimen; to test whether ctDNA clearance is associated with radiographic response, PFS, and OS; to estimate the lead time between molecular and radiographic progression; and to explore whether specific genomic alterations are associated with failure to clear ctDNA.

## 2. Materials and Methods

### 2.1. Study Population and Treatment

We retrospectively analyzed patients with histologically confirmed mUC who began first-line EV+pembro at The Ohio State University Comprehensive Cancer Center between April 2023 and August 2025. Patients were eligible if they had received at least one cycle of EV+pembro and had at least one ctDNA measurement using the Signatera tumor-informed assay (Natera, Inc., Austin, TX, USA). EV was administered at 1.25 mg/kg intravenously on days 1 and 8 of each 21-day cycle with pembrolizumab 200 mg on day 1 and dose modifications per institutional practice. Of 84 patients treated with EV+pembro at our center who were identified from an institutional database, 52 had no ctDNA testing, 1 had a sample on which the assay could not be run, and 6 had ctDNA results but did not meet the other criteria. Twenty-five patients met these criteria; their demographic, clinical, treatment, and genomic data, including metastatic sites at the start of EV+pembro, prior systemic therapy, and prior primary tumor resection, were abstracted from the electronic medical record using a predefined case report form. Because ctDNA testing was ordered at the discretion of the treating physician, the cohort is defined by the availability of testing and is not a consecutive series. Prior chemotherapy or immunotherapy for localized disease was permitted. EV+pembro was continued until disease progression. Cisplatin eligibility and subsequent therapies were not captured in the case report form.

### 2.2. ctDNA Assessment

Longitudinal ctDNA monitoring used the Signatera assay, a bespoke, tumor-informed multiplex polymerase chain reaction-based assay that tracks up to 16 patient-specific, clonal somatic variants (single-nucleotide variants and small insertions and deletions) identified from whole-exome sequencing of tumor tissue and matched normal DNA [30,31]. ctDNA was reported as the mean tumor molecules per milliliter of plasma (MTM/mL). Sampling was performed as part of routine care rather than on a protocol-defined schedule, typically every 6–12 weeks. The number of ctDNA measurements for each patient is given in Table S1. Blood was collected in Cell-Free DNA BCT tubes (Streck, La Vista, NE, USA) and shipped at ambient temperature to Natera’s clinical laboratory, where plasma processing and testing were performed; the plasma volume and processing time were not recorded at our site. Natera performed the assay as part of routine clinical care and had no role in the design, analysis, or reporting of this study. We evaluated baseline detectability (>0 MTM/mL in the baseline sample, drawn from 37 days before to the day of the first dose in 15 patients and 47, 208, and 295 days after the first dose in 3 patients), the baseline burden (the quantitative baseline value), an early decrease (any decrease from baseline within ≤60 days), ctDNA clearance (an undetectable level of 0 MTM/mL at any point during treatment—that is, ever undetectable ctDNA), and a ctDNA rise (any detectable value after an undetectable nadir or any increase above a detectable nadir; the observed magnitudes are reported in Section 3.3). In these 3 patients, no pretreatment sample was available in our data set, and an on-treatment sample was used as the baseline value. ctDNA clearance is an operational definition (reported value = 0 MTM/mL) corresponding to the assay’s reporting threshold; the analytical limit of detection follows the published Signatera validation [30,31]. All 18 patients with an evaluable baseline sample had detectable ctDNA, so an undetectable on-treatment result in these patients represents the clearance of a previously detectable signal. In the 5 patients without a baseline sample who reached 0 MTM/mL, clearance denotes an undetectable on-treatment result for which a preceding detectable value could not be documented; these patients are included in the clearance group throughout. Metrics requiring a baseline value were evaluated only in the 18 patients with an evaluable baseline sample and again in the 15 whose baseline sample was drawn before treatment; the 7 patients without a baseline draw were excluded from these analyses rather than classified as not having a decrease. The interval from ctDNA rise to progression was estimated only in patients with a ctDNA sample obtained between the nadir and the progression date.

### 2.3. Response Assessment

Response was assessed by RECIST 1.1 [16] using computed tomography or magnetic resonance imaging obtained approximately every 9–12 weeks as part of routine care. Assessments were performed by the radiologists who interpreted the images, without knowledge of the ctDNA results; responses were not centrally reviewed. The objective response rate (ORR) was the proportion of patients with a complete response (CR) or partial response (PR), and the disease control rate (DCR) was the proportion with CR, PR, or stable disease (SD).

### 2.4. Genomic Analysis

Genomic alterations were obtained from the tumor-informed Signatera workflow and supplementary clinical genomic testing (Natera Altera); all genomic results were from Natera testing. Twenty of 25 patients had genomic results; the 5 untested patients were excluded from genomic analyses and were not assumed to be wild-type. An alteration was defined as any somatic single nucleotide variant, insertion or deletion, or copy number alteration reported as pathogenic or likely pathogenic on the clinical report; variants of uncertain significance were not counted. Gene symbols were normalized to current HGNC nomenclature. *ERBB2* was analyzed as a reported genomic alteration only; HER2 protein positivity determined by immunohistochemistry was recorded separately and not pooled with *ERBB2* genomic alterations, because the two are substantially discordant in urothelial carcinoma. *TP53* was the single pre-specified gene of interest; all other genes were analyzed descriptively, as exploratory comparisons without correction for multiple testing.

### 2.5. Statistical Analysis

PFS was measured from EV+pembro initiation to documented radiographic progression or death from any cause, and OS to death from any cause, with censoring at last follow-up. Proportions were presented with exact (Clopper–Pearson) 95% CIs. Continuous variables were summarized as medians with interquartile ranges (IQRs), with quartiles computed by the inverse empirical distribution function with averaging at discontinuities. Survival was estimated by the Kaplan–Meier method, with HRs and their 95% CIs from univariate Cox proportional hazards regression. Reported *p* values for survival comparisons were based on Wald tests from the Cox models. Because ctDNA clearance is reached during treatment, comparing survival by clearance status from treatment initiation is subject to immortal time (guarantee time) bias [32]; clearance was therefore also modeled as a time-dependent covariate, with each patient contributing time to the no clearance group until the first undetectable result and to the clearance group thereafter, and the likelihood ratio test and profile likelihood CIs were used for this model. Because only one death occurred in the clearance group, Firth penalized Cox models [33] were also fitted. Log-rank *p* values are given in the Kaplan–Meier figure legend, the proportional hazards assumption was assessed with scaled Schoenfeld residuals, and the difference in restricted mean survival time at 12 months was estimated. Landmark analyses were also performed at 12 and 24 weeks, comparing patients who had and had not cleared ctDNA by the landmark among those who were event-free at that time, with time measured from the landmark. Median survival CIs were calculated using the Brookmeyer–Crowley method, and survival probability CIs were calculated using Greenwood variance with a complementary log–log transformation. Median follow-up was estimated by the reverse Kaplan–Meier method. Categorical comparisons used Fisher’s exact test; the global comparison across the four response categories used the 2 × 4 Fisher–Freeman–Halton exact test. Analyses were performed in IBM SPSS Statistics (version 31; IBM Corp., Armonk, NY, USA); Firth penalized estimates [33], profile likelihood CIs, and the restricted mean survival time were calculated with published methods; a two-sided *p* < 0.05 was considered nominally significant, and no correction was implemented for the multiple comparisons across tables.

## 3. Results

### 3.1. Patient Characteristics and ctDNA Dynamics

Twenty-five patients were included (Table 1). The median age was 69 years (range 49–85); 17 (68%) were male, and 20 (80%) had an Eastern Cooperative Oncology Group (ECOG) performance status of 0–1, while five (20%) had an ECOG performance status of 2. The primary tumor site was the bladder in 20 patients (80%) and the upper tract in five (20%). Variant histology was documented in four patients (16%: squamous differentiation in two, plasmacytoid in one, and unspecified variant histology in one). Metastatic sites were documented in 23 patients: 11 had lymph node-only disease and 12 had visceral disease, including liver metastases in five (Table 1). Fourteen patients (56%) had received chemotherapy or immunotherapy for localized disease, and five (20%) had undergone primary tumor resection. The median follow-up was 15.6 months and was similar between patients who did and did not achieve ctDNA clearance (15.9 vs. 14.4 months; *p* = 0.589). No baseline characteristic differed significantly between groups, although an ECOG performance status of 2 was more frequent (40% versus 7%), and the baseline ctDNA burden was higher among patients who did not clear ctDNA, so confounding by performance status or tumor burden cannot be excluded.

Baseline ctDNA was available for 18 patients (72%; drawn before treatment in 15) and was detectable in all 18 (100%), spanning a range (0.43–11,803 MTM/mL; median 20.8, IQR 11.6–223.8). Among these 18 patients, an on-treatment decrease occurred in 16 (89%), including 14 (78%) within 60 days; the first documented decrease was a median of 97% below baseline (range 22–100%). Overall, ctDNA clearance was achieved in 15 of 25 patients (60%; 95% CI 38.7–78.9). The median time from treatment start to the first undetectable result was 124 days (IQR 71–224; range 21–545); four patients had cleared by 12 weeks and 10 by 24 weeks, and, in at least 10 of the 15 patients, the undetectable result was confirmed on the next sample. A total of 186 ctDNA measurements were available, with a median of five per patient (range 2–20; median 10 in patients who cleared and 4.5 in those who did not; Table S1).

### 3.2. ctDNA Clearance, Response, and Disease Control

The best responses included 5 CR (20%), 14 PR (56%), 4 SD (16%), and 2 progressive disease (PD; 8%), yielding an ORR of 76% (19/25; 95% CI 54.9–90.6) and a DCR of 92% (23/25; 95% CI 74.0–99.0). The distribution of best responses differed by ctDNA status (Fisher–Freeman–Halton exact *p* = 0.003; Table 2). All 15 patients who achieved ctDNA clearance had an objective response (100%; 95% CI 78.2–100), compared with 4 of 10 (40%; 95% CI 12.2–73.8) who did not (*p* = 0.0012), and all patients with SD or PD as their best response were in the group that never cleared. The DCR was numerically higher in patients who had cleared ctDNA (100% vs. 80%), but the difference was not statistically significant (*p* = 0.150). Because the best response and ctDNA clearance were both assessed over the entire treatment course, these findings describe concordance between molecular and radiographic responses rather than demonstrating that ctDNA status predicts a subsequent scan result. Restricted to the 18 patients with an evaluable baseline, the ORR was 10 of 10 with clearance versus 3 of 8 without (*p* = 0.007), and, in the 15 patients with a pretreatment baseline sample, it was 10 of 10 versus 3 of 5 (*p* = 0.095).

### 3.3. ctDNA Dynamics and Survival

**Figure 1:**
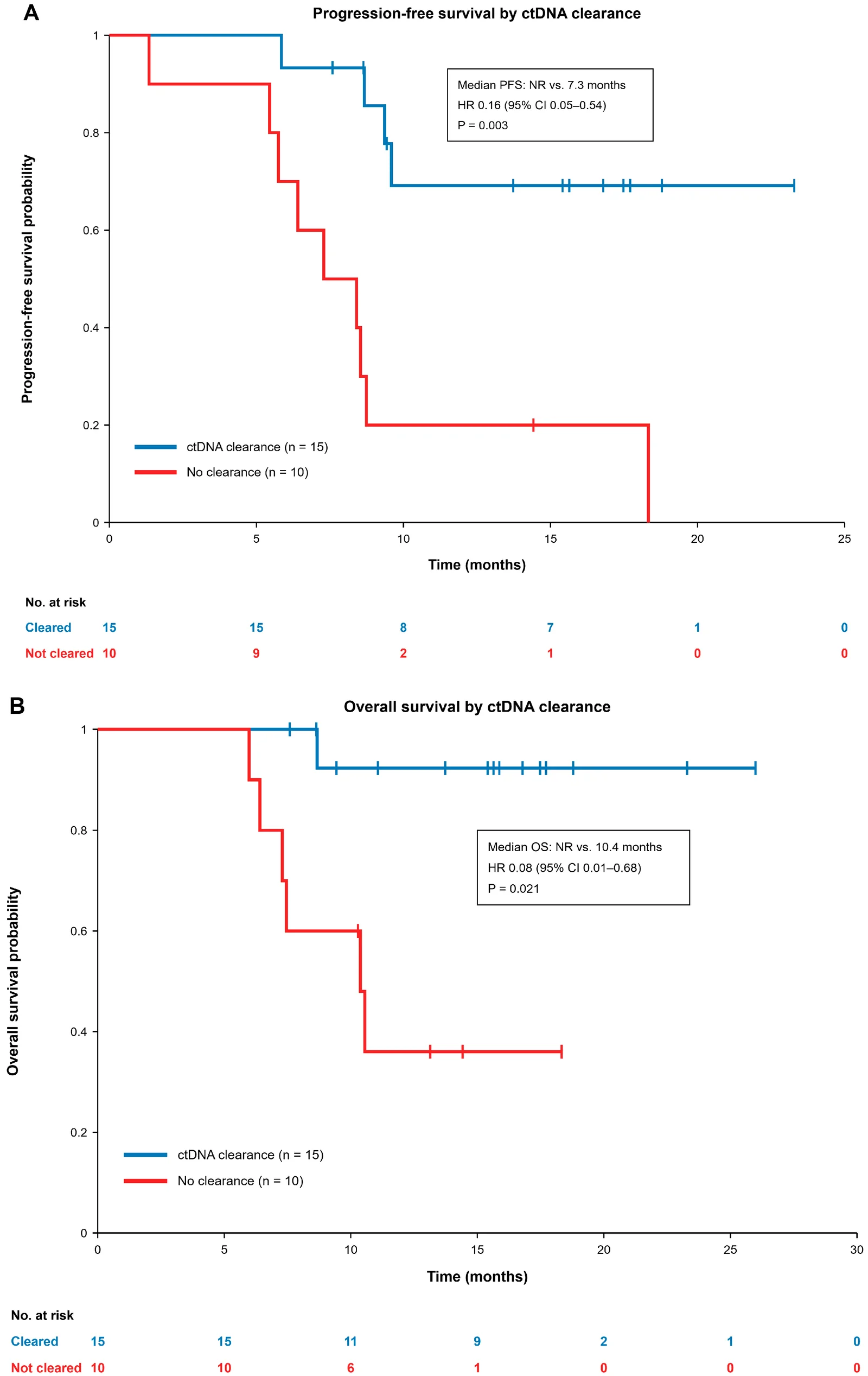
Kaplan–Meier estimates of (**A**) progression-free survival and (**B**) overall survival, stratified by ctDNA clearance. Vertical tick marks denote censored observations; curves are plotted only to the last observed time in each group. Numbers at risk are shown below each panel. HRs and *p* values in the panels are from univariate Cox regression; log-rank *p* = 0.001 (**A**) and *p* = 0.003 (**B**). Because clearance is reached during treatment, this comparison is subject to immortal time bias; the time-dependent analysis is given in Table 3.

The median PFS for the cohort was 9.6 months (95% CI 8.5–NR), and the median OS was not reached, with 13 PFS events and 7 deaths. ctDNA clearance was associated with longer PFS and OS (Figure 1, Table 3). The median PFS was not reached in patients who achieved clearance, versus 7.3 months in those who did not (HR 0.16, 95% CI 0.05–0.54; *p* = 0.003). The median OS was not reached in patients who achieved clearance, versus 10.4 months in those who did not (HR 0.08, 95% CI 0.01–0.68; *p* = 0.021). PFS events occurred in 4 of 15 patients with clearance and 9 of 10 without, and deaths occurred in 1 of 15 and 6 of 10 (log-rank *p* = 0.001 and *p* = 0.003). With clearance modeled as a time-dependent covariate, the associations were attenuated (PFS HR 0.35, 95% CI 0.09–1.15, likelihood ratio *p* = 0.085; OS HR 0.14, 95% CI 0.01–0.84, likelihood ratio *p* = 0.029; profile likelihood CIs; Table 3). Firth penalized estimates were similar to the conventional estimates (PFS HR 0.17, 95% CI 0.05–0.52; OS HR 0.11, 95% CI 0.01–0.54); the tests of proportional hazards were not significant (*p* = 0.42 for PFS and *p* = 0.75 for OS); and the differences in the restricted mean survival time at 12 months were 3.3 months for PFS (95% CI 1.2–5.4) and 2.2 months for OS (95% CI 0.6–3.7). Restricted to the 18 patients with an evaluable baseline, the estimates were similar (PFS HR 0.09, 95% CI 0.02–0.43; OS HR 0.08, 95% CI 0.01–0.69; Table 3), as they were in the 15 patients with a pretreatment baseline sample (PFS HR 0.10, 95% CI 0.02–0.53; OS HR 0.08, 95% CI 0.01–0.73). In the landmark analysis at 24 weeks, 10 patients had cleared ctDNA, and the HRs were 0.49 (95% CI 0.13–1.87) for PFS and 0.23 (95% CI 0.03–1.95) for OS (Table 3); a landmark analysis at 12 weeks was not informative because only four patients had cleared by then and none had an event. An early ctDNA decrease within 60 days, assessed among the 18 patients with an evaluable baseline, was not significantly associated with PFS (HR 0.62, 95% CI 0.16–2.36; *p* = 0.48) or OS (HR 0.65, 95% CI 0.13–3.34; *p* = 0.60) (Table 3). All 15 patients with a pretreatment baseline sample had a decrease, 14 of them within 60 days, so this comparison could not be performed in this group; three of the four patients without an early decrease among the 18 had an on-treatment baseline sample.

**Figure 2:**
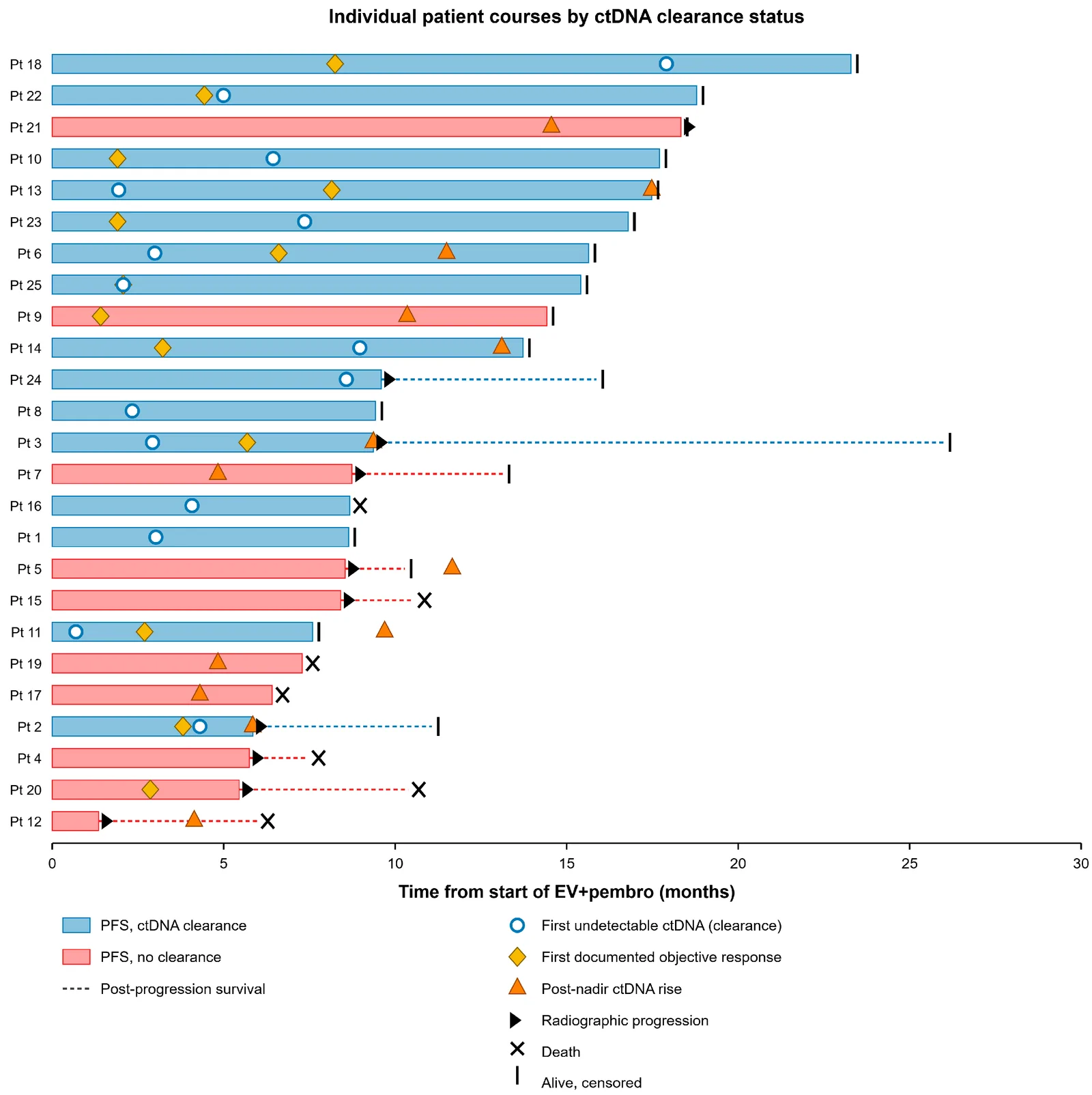
Swimmer plot of individual patient courses by ctDNA clearance status. Bars show progression-free survival and dashed lines show survival after progression. Open circles mark the first undetectable ctDNA result, diamonds mark the first documented objective response (available for 13 of 19 responders), triangles mark the first post-nadir ctDNA rise, arrowheads mark radiographic progression, crosses mark death, and vertical bars mark censoring. In patients 5 and 11, the rise was detected on a sample drawn after the last follow-up date used in the survival analysis; in patient 25, clearance and response were first documented on the same day. In patients 16, 17, and 19, the date of progression was the date of death, so only the cross is shown. The first post-nadir ctDNA rise preceded progression in patients 7, 17, 19, and 21 (the 4 patients in the lead time estimate; median 95 days); was detected on the day that progression was documented in patients 2 and 3; and was detected after progression in patients 5 and 12.

A PFS event occurred in 13 patients, comprising six with documented radiographic progression who remained alive and seven who died. Separately, a post-nadir ctDNA rise occurred in 13 patients; among the 12 patients with evaluable sampling dates, the median interval from nadir to rise was 125.5 days (range 37–473). Of the 13 patients with a post-nadir rise, nine (69%) had an objective response as their best response, eight had a PFS event, and five remained progression-free at last follow-up. A ctDNA sample obtained between the nadir and the date of progression was available in four of the eight patients who progressed, and, in these, the rise preceded progression by a median of 95 days (range 64–119); in two of these four patients, the date of progression was the date of death. In the other four, no sample was drawn between the nadir and progression: in two, the first post-nadir sample was obtained on the day that progression was documented, and in two, it was obtained only after progression, so any interval would reflect the timing of blood collection rather than the timing of the molecular rise and was not estimated. Because the rise date is the date of the first sample on which an increase was detected, these intervals are lower bounds. Of the 13 rises, six occurred after clearance (rise values 0.04–2.56 MTM/mL; two of these patients progressed) and seven in patients who never cleared (1.2 to 193 times the nadir value; six progressed). Individual patient courses are shown in Figure 2.

### 3.4. Genomic Correlates

Genomic results were available for 20 of 25 patients (Table 4). Among these 20 evaluable patients, the most frequent alterations were *TP53* (11, 55%), *ARID1A* (six, 30%), *KDM6A* (five, 25%), and *ERBB2*, *FGFR3*, *TSC1*, *RB1*, *STAG2*, and *TERT* (three each, 15%). *TP53* co-occurred with *ARID1A*, *ERBB2*, and *RB1* in three patients each and never with *FGFR3*. In the exploratory analyses (Table 3 and Table 4), *TP53* alterations were associated with a numerically lower ctDNA clearance rate (5/11, 45%, versus 8/9, 89%, in *TP53* wild type; *p* = 0.07) and showed a trend toward shorter PFS (HR 4.42, 95% CI 0.93–21.0; *p* = 0.062), without a significant association with OS (HR 1.63, 95% CI 0.30–8.95; *p* = 0.57). *ARID1A*-altered patients (*n* = 6) had an ORR of 100% (one CR, five PR), with ctDNA clearance in five of six (83%; *p* = 0.26 and *p* = 0.35 versus *ARID1A* wild type), and all three *FGFR3*-altered patients responded and cleared ctDNA (*p* = 0.54 and *p* = 0.52). Among the three patients with a reported *ERBB2* genomic alteration, two responded (67%) and one cleared ctDNA (33%). One additional patient had HER2 protein positivity by immunohistochemistry without a reported *ERBB2* alteration and was analyzed separately. *TSC1* alterations (*n* = 3) showed a non-significant trend toward shorter PFS (HR 3.64, 95% CI 0.61–21.9; *p* = 0.16). All genomic comparisons involved small subgroups, were confounded by co-occurring alterations, and were not corrected for multiple testing; none reached statistical significance.

## 4. Discussion

In this cohort of 25 patients with metastatic urothelial carcinoma, ctDNA clearance identified a group in which every patient responded radiographically and in which both PFS and OS were longer. Three features distinguish this analysis from the ctDNA literature in this disease. First, unlike previously reported series, which included both EV monotherapy and the combination or spanned several lines of therapy, our cohort was restricted to patients receiving first-line EV+pembro. The kinetics described here were therefore observed under a single contemporary standard-of-care regimen. Second, ctDNA clearance was associated not only with progression-free survival but also with overall survival (HR 0.08, 95% CI 0.01–0.68), consistent with the association reported in a mixed EV with or without pembrolizumab cohort [25], although the associations were attenuated when clearance was modeled as a time-dependent covariate. Third, in the exploratory analyses, clearance and response differed numerically by genomic context: *TP53*-altered tumors cleared ctDNA less often, whereas *ARID1A*- and *FGFR3*-altered tumors were over-represented among responders, although no comparison reached statistical significance. Because the cohort is single-arm, these relationships are prognostic and response-associated rather than predictive of a benefit from EV+pembro specifically, a distinction that would require a randomized comparison.

Our results are consistent with and extend the existing ctDNA data in EV-treated urothelial carcinoma. In the largest series reported to date, Reyes and colleagues analyzed 545 plasma samples from 110 patients receiving EV with or without pembrolizumab and found baseline ctDNA to be detectable in 93.6%, a decline within 12 weeks in 75.8%, clearance within 12 weeks in 33.9%, and inferior progression-free and overall survival with on-treatment ctDNA positivity at every interval examined [25]. Their earlier report of 36 patients described a 91% response rate among the 11 patients who cleared ctDNA and a median of 124 days from ctDNA rise to radiographic progression [29]. Our observations were concordant in direction: an on-treatment decrease occurred in 89% of evaluable patients, 60% achieved ctDNA clearance, and every patient who cleared responded radiographically. The higher clearance rate in our series is most plausibly explained by ascertainment at any point during treatment rather than within a fixed 12-week window (only 4 of our 25 patients had cleared by 12 weeks), together with restriction to combination therapy. In the earlier report of 36 patients from that series, the survival advantage associated with clearance did not reach statistical significance for overall survival [29], whereas the full analysis of 110 patients found an association with both endpoints [25], as did our uniformly combination-treated cohort.

Two further real-world series support the same conclusion. Aslanova and colleagues followed 47 patients with locally advanced or metastatic urothelial carcinoma and reported that failure to convert a positive baseline ctDNA result to negative portended a poor prognosis, with mortality reaching 70% among patients with metastatic disease and persistent baseline positivity [27]. Karol and colleagues, analyzing 48 pre-treatment timepoints from 38 patients, showed that the quantitative baseline ctDNA level carries prognostic information beyond simple detectability, with a fully adjusted hazard ratio for death of 3.0 (95% CI 1.5–5.8) per log10 increase and superior discrimination relative to a binary detectability threshold [26]. The pretreatment tumor burden and on-treatment clearance may each add prognostic information. In our cohort, the baseline burden was numerically higher in patients who never cleared (median 109.4 versus 16.5 MTM/mL; the baseline value was an on-treatment sample in three of the eight patients), but the difference was not statistically significant among the 18 patients with an evaluable baseline, and the study was not powered to model baseline ctDNA as a continuous variable. A practical monitoring strategy could pair a quantitative pretreatment measurement for risk stratification with serial on-treatment testing for response assessment.

A post-nadir ctDNA rise preceded progression by a median of 95 days in the four patients in whom this interval could be measured, and the median interval from nadir to molecular rise was 125.5 days. This lead time is consistent with the 124 days from ctDNA rise to progression reported in the earlier EV with or without pembrolizumab series [29] and with the observation that ctDNA identifies relapse earlier than imaging alone [27]; the interval from nadir to rise (125.5 days) is a different quantity, and its similarity to 124 days is coincidental. A lead time of roughly three to four months, if confirmed, might allow imaging to be timed earlier in patients with a rise, but our data do not support changing treatment on the basis of a rise alone. This estimate remains preliminary: it rests on four patients, in two of whom the date of progression was the date of death, and, because blood sampling was not protocol-defined, the interval could not be computed for every patient who progressed. Prospective studies with fixed assessment intervals are required to validate this estimate.

The genomic correlates are exploratory, and none reached statistical significance. *TP53*-altered tumors cleared ctDNA less often (45% versus 89%) and trended toward shorter progression-free survival, consistent with the association of *TP53* alteration with genomic instability and aggressive behavior in urothelial carcinoma [34,35]. Conversely, all six *ARID1A*-altered and all three *FGFR3*-altered patients responded. *ARID1A*, a subunit of the SWI/SNF chromatin-remodeling complex, is among the most frequent alterations in urothelial carcinoma and has been linked to immune-inflamed phenotypes, whereas *FGFR3* alteration defines a luminal-papillary subtype often regarded as less immune-responsive [36,37,38]. By contrast, an earlier real-world ctDNA series found no correlation between outcome and next-generation sequencing findings [27]. With three to six patients per subgroup and extensive co-mutations, these observations are hypothesis-generating and require validation.

Until prospective data are available, the following suggestions are speculative and were not tested in this study. A persistent failure to clear ctDNA, or a rise after clearance, could be a reason to obtain the next scan at the earlier end of the usual interval, whereas undetectable ctDNA would not justify more frequent imaging. During pembrolizumab therapy, a falling or undetectable ctDNA level at the time of equivocal radiographic growth might favor continuing treatment and repeating imaging rather than assuming true progression [17]. A ctDNA rise alone should not prompt a change in therapy: 5 of the 13 rises in our cohort were not followed by progression during follow-up, although follow-up after the rise was short or absent in three of them. Progression by RECIST should remain the standard for discontinuation.

This study has several limitations. Its retrospective, single-center design and small sample size (*n* = 25), with few survival events, limit the statistical power and preclude multivariable adjustment; accordingly, the reported HRs have wide CIs and should be considered hypothesis-generating. ctDNA clearance was ascertained at any point during treatment rather than at a fixed landmark, so patients with longer treatment exposure and more ctDNA measurements (median 10 versus 4.5 in those who did not clear) had a greater opportunity to be classified as having cleared, and the associations were attenuated when clearance was modeled as a time-dependent covariate; likewise, the best response and ctDNA status were both assessed over the entire course, so their association reflects concordance rather than a prediction. The ORR of 76% (95% CI 54.9–90.6) is compatible with the confirmed ORR of about 68% in EV-302 [13]; the median PFS, which was shorter than in that trial, may reflect the inclusion of patients with an ECOG performance status of 2 (20%). The cohort was defined by the availability of ctDNA testing and is not a consecutive series, and an ECOG performance status of 2 was more frequent among patients who had never cleared, so confounding by performance status cannot be excluded. Cisplatin eligibility and subsequent therapies were not captured. An early ctDNA decrease within 60 days was not significantly associated with PFS or OS after the analysis was restricted to the 18 patients with an evaluable baseline sample, and we therefore do not regard an early decrease as an established prognostic marker in this cohort; a transient rise in ctDNA shortly after the start of treatment (flare) may also have masked an early decrease. Baseline ctDNA was unavailable for seven patients, which, together with the small sample size, is the main reason that the quantitative baseline burden could not be related to response, disease control, PFS, or OS with useful precision; the timing and frequency of ctDNA sampling were also not standardized, which affects the time-to-nadir and lead-time estimates. The tumor-informed assay tracks variants identified in the primary tumor and may not capture metastatic subclonal evolution [39]. Finally, more than 20 comparisons were performed across Table 1, Table 2, Table 3 and Table 4 without formal correction.

## 5. Conclusions

In patients with mUC treated with first-line EV+pembro, achieving ctDNA clearance was associated with radiographic response and with longer progression-free and overall survival, although the survival association was attenuated after accounting for the time needed to achieve clearance. A post-nadir ctDNA rise preceded progression in a subset of patients. These findings, from a small single-arm retrospective cohort, are hypothesis-generating and support the prospective, protocol-defined evaluation of ctDNA-guided monitoring in this setting.

## Figures and Tables

**Table 1:** Demographics and clinical characteristics, stratified by ctDNA clearance.

	ctDNA Clearance (*n* = 15)	No Clearance (*n* = 10)	*p* Value	All Patients (*N* = 25)
Age (median, IQR), years	70 (62–78)	66.5 (63–74)	0.542	69 (63–75)
Sex, *n* (%)			0.667	
Male	11 (73.3)	6 (60.0)		17 (68.0)
Female	4 (26.7)	4 (40.0)		8 (32.0)
ECOG performance status, *n* (%)			0.121	
0–1	14 (93.3)	6 (60.0)		20 (80.0)
2	1 (6.7)	4 (40.0)		5 (20.0)
Primary tumor site, *n* (%)			0.615	
Bladder	11 (73.3)	9 (90.0)		20 (80.0)
Upper tract	4 (26.7)	1 (10.0)		5 (20.0)
Histology, *n* (%)			0.626	
Pure urothelial	12 (80.0)	9 (90.0)		21 (84.0)
Variant	3 (20.0)	1 (10.0)		4 (16.0)
Prior systemic therapy for localized disease, *n* (%)	9 (60.0)	5 (50.0)	0.697	14 (56.0)
Prior primary tumor resection, *n* (%)	1 (6.7)	4 (40.0)	0.121	5 (20.0)
Metastatic sites, *n* (%) ^1^				
Lymph node only	7 (53.8)	4 (40.0)	0.680	11 (47.8)
Visceral, any	6 (46.2)	6 (60.0)	0.680	12 (52.2)
Liver	3 (23.1)	2 (20.0)	1.000	5 (21.7)
Baseline ctDNA available, *n* (%)	10 (66.7)	8 (80.0)	0.659	18 (72.0)
Baseline ctDNA (median, IQR), MTM/mL ^2^	16.5 (11.6–79.3)	109.4 (13.9–1585.9)	0.248	20.8 (11.6–223.8)
Median follow-up, months	15.9	14.4	0.589	15.6

ctDNA, circulating tumor DNA; ECOG, Eastern Cooperative Oncology Group; IQR, interquartile range; MTM/mL, mean tumor molecules per milliliter. *p* values are from Fisher’s exact test for categorical variables, the Mann–Whitney U test for age and baseline ctDNA, and the log-rank test on reverse Kaplan–Meier curves for follow-up. ^1^ Documented in 23 patients (13 with clearance, 10 without); visceral disease includes bone, liver, lung, and peritoneal metastases. ^2^ Among patients with a baseline sample (*n* = 10 with clearance, *n* = 8 without; in 3 of the 8, the baseline value was an on-treatment sample).

**Table 2:** Best radiographic response and disease control by ctDNA clearance.

Best Response	ctDNA Clearance (*n* = 15)	No Clearance (*n* = 10)	All Patients (*N* = 25)
Complete response, *n* (%)	4 (26.7)	1 (10.0)	5 (20.0)
Partial response, *n* (%)	11 (73.3)	3 (30.0)	14 (56.0)
Stable disease, *n* (%)	0 (0)	4 (40.0)	4 (16.0)
Progressive disease, *n* (%)	0 (0)	2 (20.0)	2 (8.0)
Global test across the four response categories	*p* = 0.003		
Objective response rate (CR + PR), *n* (%)	15 (100)	4 (40.0)	19 (76.0)
ORR, ctDNA clearance vs. no clearance	*p* = 0.0012		
Disease control rate (CR + PR + SD), *n* (%)	15 (100)	8 (80.0)	23 (92.0)
DCR, ctDNA clearance vs. no clearance	*p* = 0.150		

Responses were assessed by radiologists blinded to ctDNA results. CI, confidence interval; CR, complete response; ctDNA, circulating tumor DNA; DCR, disease control rate; ORR, objective response rate; PR, partial response; SD, stable disease.

**Table 3:** Univariate Cox regression analyses for progression-free survival (PFS) and overall survival (OS).

Variable	Analysis *N* (PFS Events/Deaths)	PFS HR (95% CI)	*p*	OS HR (95% CI)	*p*
ctDNA clearance (achieved vs. not)	25 (13/7; 4 vs. 9/1 vs. 6)	0.16 (0.05–0.54)	0.003	0.08 (0.01–0.68)	0.021
ctDNA clearance as a time-dependent covariate	25 (13/7; 4 vs. 9/1 vs. 6)	0.35 (0.09–1.15)	0.085 ^1^	0.14 (0.01–0.84)	0.029 ^1^
ctDNA clearance, patients with evaluable baseline	18 (11/7; 3 vs. 8/1 vs. 6)	0.09 (0.02–0.43)	0.003	0.08 (0.01–0.69)	0.022
ctDNA clearance by 24 weeks, landmark analysis ^2^	23/25 (11/7; 3 vs. 8/1 vs. 6)	0.49 (0.13–1.87)	0.30	0.23 (0.03–1.95)	0.18
Early ctDNA decrease ≤60 days (vs. not)	18 (11/7; 8 vs. 3/5 vs. 2)	0.62 (0.16–2.36)	0.48	0.65 (0.13–3.34)	0.60
*TP53* alteration (vs. wild type)	20 (10/6; 8 vs. 2/4 vs. 2)	4.42 (0.93–21.0)	0.062	1.63 (0.30–8.95)	0.57
*TSC1* alteration (vs. wild type)	20 (10/6; 2 vs. 8/1 vs. 5)	3.64 (0.61–21.9)	0.16	1.63 (0.19–14.2)	0.66

CI, confidence interval; ctDNA, circulating tumor DNA; HR, hazard ratio; OS, overall survival; PFS, progression-free survival. Events are given for the group named in each row vs. its comparison group. Firth penalized estimates for ctDNA clearance were 0.17 (0.05–0.52) for PFS and 0.11 (0.01–0.54) for OS. ^1^ Likelihood ratio test; events are counted in the state occupied at the time of the event. CIs are profile likelihood intervals, which agree with the likelihood ratio test; the Wald 95% CIs were 0.10–1.21 for PFS and 0.02–1.18 for OS. ^2^ Patients who were event-free at 24 weeks (23 for PFS, 25 for OS), compared by clearance status at that time, with time measured from the landmark.

**Table 4:** Recurrent genomic alterations and exploratory correlates among the 20 genomically evaluable patients.

Alteration	*n* (% of 20 Evaluable)	ORR (CR + PR) ^1^	ctDNA Clearance Rate ^1^
*TP53*	11 (55)	7/11 (64%); *p* = 0.32	5/11 (45%); *p* = 0.07
*ARID1A*	6 (30)	6/6 (100%); *p* = 0.26	5/6 (83%); *p* = 0.35
*KDM6A*	5 (25)	3/5 (60%); *p* = 0.56	3/5 (60%); *p* = 1.00
*ERBB2* (genomic alteration)	3 (15)	2/3 (67%); *p* = 1.00	1/3 (33%); *p* = 0.27
*FGFR3*	3 (15)	3/3 (100%); *p* = 0.54	3/3 (100%); *p* = 0.52
*TSC1*	3 (15)	3/3 (100%); *p* = 0.54	2/3 (67%); *p* = 1.00
*RB1*	3 (15)	2/3 (67%); *p* = 1.00	2/3 (67%); *p* = 1.00
*STAG2*	3 (15)	2/3 (67%); *p* = 1.00	2/3 (67%); *p* = 1.00
*TERT*	3 (15)	2/3 (67%); *p* = 1.00	2/3 (67%); *p* = 1.00

^1^ Fisher’s exact test, altered versus wild type among the 20 evaluable patients; no correction for multiple testing. CR, complete response; ctDNA, circulating tumor DNA; ORR, objective response rate; PR, partial response.

## Data Availability

The de-identified data supporting the findings of this study are available from the corresponding author upon reasonable request. The data are not publicly available because they contain information that could compromise the privacy of the research participants.

## Supplementary Materials

The following supporting information can be downloaded at: https://www.mdpi.com/article/10.3390/cancers18213532/s1, Table S1: Number of ctDNA measurements per patient.

## Abbreviations

The following abbreviations are used in this manuscript:

CI	Confidence interval
CR	Complete response
ctDNA	Circulating tumor DNA
DCR	Disease control rate
DFS	Disease-free survival
ECOG	Eastern Cooperative Oncology Group
EV	Enfortumab vedotin
EV+pembro	Enfortumab vedotin plus pembrolizumab
FDA	U.S. Food and Drug Administration
HER2	Human epidermal growth factor receptor 2
HGNC	HUGO Gene Nomenclature Committee
HR	Hazard ratio
IQR	Interquartile range
MTM/mL	Mean tumor molecules per milliliter
mUC	Metastatic urothelial carcinoma
NR	Not reached
ORR	Objective response rate
OS	Overall survival
PD	Progressive disease
PD-1	Programmed cell death protein 1
PD-L1	Programmed death ligand 1
PFS	Progression-free survival
PR	Partial response
RECIST	Response Evaluation Criteria in Solid Tumors
SD	Stable disease
