# Supplementary material for: Circulating Tumor DNA Clearance Is Associated with Response and Survival in Metastatic Urothelial Carcinoma Treated with First-Line Enfortumab Vedotin Plus Pembrolizumab
Source: Cancers (Basel). 2026 Nov 3;18(21):3532. doi: 10.3390/cancers18213532 (PMC13761457; doi:10.3390/cancers18213532)
Supplement: Supplementary file 1 [file cancers-18-03532-s001.zip › cancers-4557272-supplementary.pdf]

Supplementary Materials

Circulating Tumor DNA Clearance Is Associated with Response and Survival in Metastatic Urothelial Carcinoma Treated with First-Line Enfortumab Vedotin plus Pembrolizumab

Table S1. Number of ctDNA measurements per patient.

**Patient**	**ctDNA clearance**	**Number of ctDNA measurements**
1	Yes	5
2	Yes	11
3	Yes	20
4	No	2
5	No	5
6	Yes	18
7	No	8
8	Yes	10
9	No	4
10	Yes	12
11	Yes	14
12	No	4
13	Yes	2
14	Yes	12
15	No	2
16	Yes	4
17	No	7
18	Yes	3
19	No	5
20	No	7
21	No	3
22	Yes	9
23	Yes	11
24	Yes	3
25	Yes	5
**All patients**		186 (median 5, range 2–20)

ctDNA, circulating tumor DNA. Measurements were counted from the Signatera reports issued between April 2023 and August 2025.
